# Management of High-Risk Localized Prostate Cancer

**DOI:** 10.1155/2012/641689

**Published:** 2011-11-01

**Authors:** Ariel E. Marciscano, Matthew E. Hardee, Nicholas Sanfilippo

**Affiliations:** Department of Radiation Oncology, NYU School of Medicine, New York, NY 10016, USA

## Abstract

Traditionally, patients with high-risk localized prostate cancer have been an extremely challenging group to manage due to a significant likelihood of treatment failure and prostate cancer-specific mortality (PCSM). The results of multiple large, prospective, randomized trials have demonstrated that men with high-risk features who are treated in a multimodal fashion at the time of initial diagnosis have improved overall survival. Advances in local treatments such as dose-escalated radiotherapy in conjunction with androgen suppression and postprostatectomy adjuvant radiotherapy have also demonstrated benefits to this subset of patients. However, therapeutic enhancement with the addition of chemotherapy to the primary treatment regimen may help achieve optimal disease control.

## 1. Introduction

Prostate cancer is the most common noncutaneous malignancy and is the second leading cause of cancer-related mortality among men in the USA [1]. In 2010, it is estimated that 217,730 men were newly diagnosed and 32,050 men died of prostate cancer [2]. Simply stated, roughly 1 in 6 American men will be confronted with a diagnosis of prostate cancer during their lifetime. Prostate cancer exhibits a broad spectrum of clinical behaviors, ranging from microscopic, well-differentiated indolent tumors to aggressive malignancies with significant potential for recurrence and metastasis. Most prostate cancers are localized at the time of diagnosis which is likely to continue with increasing emphasis on screening and improving technology for early detection. 

Historically, patients with localized prostate cancer were categorized primarily based on clinical staging and whether or not they were considered surgical candidates. Thus, the term “localized” generally referred to stage T1-T2 disease which was managed with local therapy (surgery, radiotherapy) or active surveillance. “Locally advanced” disease referred to stage T3-T4 disease which was considered inoperable. However, a better understanding of the natural history of prostate cancer and advances in both the quality and quantity of available treatment options have allowed clinicians to develop more sophisticated risk stratification systems (Table 1).

Current risk stratification of prostate cancer patients is based upon the likelihood of recurrence after locoregional treatment (Table 2). Various pretreatment parameters have been studied as potential prognostic factors to help identify subsets of patients, specifically, among high-risk patients in whom treatment failure is more likely. Prostate-specific antigen (PSA) has been one of the most extensively studied parameters (PSA velocity, PSA doubling time) but remains a source of controversy, particularly with regards to its utility in screening at-risk patients. While PSA can provide general information about the aggressiveness of the tumor or treatment response of a patient's disease, its predictive value alone remains relatively low. The incorporation of pre-treatment PSA and the Gleason score in combination with clinical staging has served to better prognosticate patient outcomes. In a multi-institutional study of 4133 prostate cancer patients, combining preoperative serum PSA levels with the Gleason score and clinical stage was able to more accurately predict capsular penetration, involvement of seminal vesicles and pelvic lymph nodes [3]. Several prior studies have confirmed that PSA > 10 ng/mL and/or Gleason scores > 7 result in a 5-year recurrence risk of approximately 70%. Furthermore, men with high-risk features at presentation have a significantly higher risk of recurrence, metastatic disease and prostate-cancer-related mortality [4–10]. 

A recent European multi-institutional study by Spahn et al. highlighted the need to further define the high-risk population in order to deliver the most appropriate therapy to each patient. In this study, 712 high-risk patients with PSA > 20 ng/mL underwent radical prostatectomy with bilateral pelvic lymph node dissection between 1987 and 2005. Patients were stratified into four subgroups based on the number of additional risk factors present (none, biopsy Gleason score ≥ 8, clinical stage 3-4, or both) to assess which risk factors improved prediction of treatment failure and PCSM. The biopsy Gleason score was the strongest predictor of progression and mortality. Among high-risk patients with PSA > 20 ng/mL, those with Gleason scores < 8 had a 10-year PCSM of 5%, while those with Gleason scores ≥ 8 had a PCSM of 35%. Importantly, this study reports that men with PSA > 20 ng/mL and a Gleason score < 8 are at minimal risk for PCSM and may represent a specific subgroup of high-risk patients that should be considered for surgery [11]. 

Similarly, a retrospective study by Walz et al. reported that the number of risk factors (T3 disease, Gleason ≥ 8, D'Amico high-risk group, PSA ≥ 20 ng/mL) present influences the 5-year biochemical recurrence risk in the postradical prostatectomy setting. The rate of favorable pathology and recurrence after surgical intervention is dependent upon the criteria used to define high-risk disease as well as the conglomeration of risk factors present in each patient [12]. Taken altogether, this suggests that there is still much to learn about high-risk disease and that further characterization of this risk group is necessary in order to optimize treatment.

The most current guidelines define high-risk localized prostate cancer as patients with clinical stage T3 disease, a Gleason score of 8–10 or a PSA level > 20 ng/mL (Table 3). Additionally, the National Comprehensive Cancer Network (NCCN) has defined very high-risk (locally advanced) patients as those with clinical stage T3b and T4 disease without evidence of nodal or metastatic involvement [13–17]. For the purposes of this paper and the discussion of therapeutic management, both high-risk and very high-risk subgroups will be considered together. While no consensus exists with regards to optimal treatment for this subset of patients, it is clear that a multidisciplinary and multimodal therapeutic approach is crucial for proper management of high-risk localized prostate cancer. This paper will focus upon current treatment modalities as well as therapeutic options on the horizon for high-risk patients.

## 2. Rationale for a Multimodal Approach for the Treatment of High-Risk Prostate Cancer

Traditionally, single modality regimens for treating high-risk patients have resulted in poor treatment responses and high failure rates [18]. These poor clinical outcomes are observed irrespective of the primary treatment type, either a surgical approach with radical prostatectomy (RP) or radiotherapy with external-beam radiation therapy (EBRT) or brachytherapy. A study by Pisansky et al., assessed disease relapse in 500 patients with clinically localized prostate cancer treated solely with radiotherapy. The total RT dose administered was dependent on tumor stage: T1 received a median dose of 64 Gy (range 60–70.7 Gy); T2 64.8 Gy (range, 50–70.2 Gy); T3-4, 66.3 Gy (range, 55.8–70.4 Gy). Amongst high-risk patients, a 24% relapse-free probability at 5 years as well as a much higher incidence of clinical and biochemical relapse was reported when compared to their low and intermediate risk counterparts [19]. Furthermore, a 2005 multi-institutional review by Soloway and Roach further delineated the need to improve existing therapeutic interventions. High-risk patients undergoing monotherapy with curative intent with RP, EBRT, or brachytherapy had high rates of both clinical and biochemical progressions (>50% at 5 years). Importantly, this paper also alluded to the increasing importance of adjuvant therapy and multimodal approaches in order to improve control of high-risk localized disease [20].

## 3. Current Recommendations for Management of High-Risk Prostate Cancer

### 3.1. Radiotherapy as a Treatment Option in High-Risk Prostate Cancer

Technological advances in existing treatment modalities and the combination of local and hormonal therapies have led to considerable progress in disease control and survival outcomes. Radiotherapy has been and will continue to be a key component in the treatment of prostate cancer and much effort has been dedicated to increasing its therapeutic efficacy with new techniques to deliver radiation. The advent of three-dimensional conformal radiation therapy (3D-CRT) and intensity-modulated radiation therapy (IMRT) have allowed radiation oncologists to achieve safe dose escalation while limiting local tissue toxicities classically associated with EBRT, such as genitourinary and bowel complications. Several independent studies have confirmed that dose escalation is associated with improved biochemical outcomes in addition to a lower risk of radiation-associated late side effects [21–25]. A pivotal randomized control trial by Kuban et al. at M.D. Anderson Cancer Center served to validate doses as high as 75–80 Gy as a permissible dose escalation for the high-risk cohort. Among the 301 patients enrolled in this long-term study, the subset of patients with adverse prognostic features (PSA > 10 ng/mL at diagnosis) derived the greatest benefit at a dose of 78 Gy compared to 70 Gy, in terms of biochemical and clinical failure [26]. Furthermore, with increasing doses, accurate and precise delivery of radiation using technology such as image-guided radiation therapy (IGRT) becomes even more important to avoid normal tissue toxicities. IGRT should also be considered in order to compensate for changes in target volume as the tumor shrinks. 

Recently, both volumetric-modulated arc therapy (VMAT) and helical tomotherapy techniques have been evaluated as novel ways to deliver radiotherapy. Several preliminary studies have evaluated the dosimetric feasibility of treating a broad spectrum of prostate cancer with VMAT, including localized, locally advanced and postoperative disease [27, 28]. A retrospective review of 292 patients treated with a VMAT method to a dose of 77.4 Gy was compared to a fixed-angle, 7-field IMRT technique using the same planning datasets and contours. It was reported that VMAT therapy resulted in a lower dose of delivery to critical structures such as the penile bulb, bladder, and femoral heads, particularly in high-dose regions with comparable dose delivery to target volumes when compared to IMRT. These results suggest further evaluation of VMAT in order to reduce radiotherapy-related acute and chronic toxicities [29]. Similar dosimetric studies evaluating the ability of helical tomotherapy to improve dose conformity and normal tissue sparing in comparison to IMRT have demonstrated overall improvement in critical organ sparing as well as achieving better homogeneity of dose delivery [30]. Of note, the improving precision and dose escalation in the administration of radiotherapy to target volumes has lead to the development of simultaneous integrated boost radiotherapy, which delivers different doses per fraction to different target regions of interest. Consequently, whole pelvis radiation therapy, which has traditionally been a controversial issue, is now a valid therapeutic consideration for node-positive or high-risk patients because high doses can be delivered to a focal target volume while also administering a lower dose of RT to the rest of the pelvis which may promote locoregional control and address micrometastatic disease [31].

### 3.2. Androgen Deprivation Therapy (ADT) in Combination with Radiotherapy

Arguably, combination therapy with radiation and long-term androgen deprivation therapy (ADT) has been one of the most important modifications to modern clinical practice for prostate cancer. The rationale of the combined approach is that the addition of ADT is believed to slow progression of the tumor by eliminating the hormonal stimulus that drives cancer cell proliferation [32]. Furthermore, in vivo animal models have shown that the combined effect of ADT and radiotherapy increases overall cell kill and diminishes growth velocity of the surviving cancer cells [33, 34]. Several agents, such as luteinizing hormone-releasing hormone (LHRH) analogs and nonsteroidal antiandrogens have been used to exploit the sensitivity of prostate cancer to hormonal suppression. Of note, surgical (bilateral orchiectomy) and medical castration are of equal efficacy and using multiple methods of androgen blockade does not confer an additive benefit for nonmetastatic patients [35]. 

Several prospective studies have demonstrated that the combination of radiotherapy and long-term androgen suppression improves disease control and survival, compared with either treatment alone for men with adverse risk factors. A prospective phase III trial, EORTC 22863, enrolled 415 men randomized to either radiotherapy alone or radiotherapy plus three years of LHRH analog (goserelin) to assess the additive effect long-term ADT in locally advanced patients. The study reported an increase in both disease-free and overall survival in the combination therapy group. Furthermore, the 10-year results of this study found no increase in cardiovascular toxicity in addition to the survival benefit [36–38]. In 2009, a prospective randomized study by Widmark et al. involving 875 high-risk patients (stage T3, PSA < 70 ng/mL) receiving ADT monotherapy (total androgen blockade for 3 months followed by continuous flutamide 250 mg) or ADT with radiotherapy also demonstrated a survival benefit with a minimal yet acceptable increase in side effects in the combination therapy cohort [39]. Similar findings were reported by D'Amico et al. in 2008, who reported an overall survival benefit in high-risk patients receiving combination therapy, despite a shorter course of ADT treatment than the aforementioned studies [40]. 

The optimal duration of ADT has been a controversial topic, and several studies have examined whether or not the long-term side effects of ADT outweigh the clinical benefits. It is understood that the incidence of side effects correlates with the duration of ADT treatment. Long-term complications associated with ADT are both real and severe, ranging from osteoporosis with risk of pathologic fracture, metabolic dysfunction including development of diabetes as well as cardiovascular disease with potential for fatal myocardial infarction [41, 42]. In 2009, Bolla et al. reported the results of the prospective EORTC 22961 trial randomizing 885 men with T2c-T4, N0 disease, in order to assess whether short-term ADT (6 months) was able to achieve the same survival benefits that had previously been reported with long-term ADT (≥2 years) while simultaneously reducing exposure to hormonal therapy. The 5-year overall mortality rate was 19.0% and 15.2% for short-term and long-term ADT, respectively; demonstrating that short-term ADT in conjunction with radiotherapy is inferior with regards to overall survival [43]. Furthermore, RTOG 9202, a large phase III trial of 1554 patients with T2c-T4 non-metastatic high-risk disease, reported that long-term ADT with radiotherapy is superior to short-term ADT with regards to disease-free survival, risk of distant metastasis, local progression and incidence of biochemical failure; however, no difference in overall survival was observed. A criticism of this study by Horwitz and colleagues is that it was not sufficiently powered to assess for overall survival. Upon subset analysis of 337 patients with a Gleason score 8–10 a benefit in overall survival was seen in the long-term ADT cohort [44]. A Canadian multicenter phase III trial examining short-versus long-term neoadjuvant ADT in combination with radiotherapy found that increasing the duration of neoadjuvant ADT from 3 to 8 months conferred a significant disease-free survival benefit among high-risk patients (42% versus 71% 5-year disease-free survival rate) [45]. While the duration of ADT has definitively demonstrated an effect on patient survival, the sequencing (adjuvant, concurrent, neoadjuvant) of when ADT is administered in relation to radiotherapy does not appear to affect outcomes in men with high-risk prostate cancer [46–48].

In summary, the current standard of care for high-risk and locally advanced disease is EBRT in conjunction with long-term ADT; specifically, a 3D-CRT or IMRT radiation therapy technique to a dose of 75–80 Gy in conjunction with long-term ADT in a neoadjuvant, concurrent, or adjuvant setting for approximately 2-3 years. Generally, high-risk patients are usually not considered for treatment with brachytherapy; however, certain clinical scenarios may warrant the use of brachytherapy boost in combination with EBRT, with consideration of short-term ADT [49]. Additionally, a surgical approach may be considered for selected high-risk-patients, although, it is a seemingly less popular approach due to the invasive nature in comparison to EBRT as well as the distinct set of complications which surgery poses; including perioperative mortality, long-term sexual dysfunction, and urinary incontinence. Additionally, the high likelihood that postoperative radiotherapy will be required potentially exposes patients to toxicities of both surgery and radiotherapy. 

### 3.3. Surgery as a Treatment Option for High-Risk Prostate Cancer

Like radiotherapy, as technology (i.e., laparoscopic, robotic) continues to improve, some of the issues with surgical management for high-risk patients are no longer valid. For example, with the advent of robotic surgery some experienced urologists now consider stage T3a prostate cancer as an operable disease. Men with clinically localized tumors without fixation that can be completely excised may be candidates for radical prostatectomy (RP) with pelvic lymph node dissection if they have a reasonable life expectancy. Lau et al., reported a post-RP overall survival of 67% at 10 years among patients with adverse prognostic features (Gleason score ≥ 8), suggesting that radical prostatectomy may be a viable alternative for patients who are not candidates for radiotherapy or whom prefer surgery [50]. 

Additionally, two recent studies have shown that surgical intervention in the high-risk cohort may result in superior clinical and survival outcomes. A 2010 retrospective study by Zelefsky et al., reported that high-risk patients undergoing RP had a lower risk of metastatic progression and PCSM compared to patients receiving IMRT (≥81 Gy) [51]. High-risk disease was defined as clinical stage T3, Gleason score 8–10, or PSA > 20 ng/mL; within this subgroup those patients treated with RP with bilateral lymphadenectomy had a 7.8% decrease in 8-year metastatic progression compared to those treated with RT (hazard ratio, 0.35). Furthermore, a 2010 retrospective analysis by Abdollah et al. examined survival outcomes patients treated with RP, RT, or observation between 1988 and 2006 and noted favorable survival rates in most patients undergoing RP [52]. Amongst high-risk patients (T2c and/or Gleason score 8–10), patients ≤ 69 years of age treated with RP derived the greatest survival benefit (PCSM 5.8–7.2%) in comparison to those treated with RT or observed (PCSM 9.9–11.3% and 21.5–21.9%, resp.). However, among patients older than 70 years of age, treatment with RT was associated with a lower PCSM (12.2–21.1%) when compared to those treated with RP or observation (PCSM = 12.2–21.1%, 18.5–19.8%, resp.). 

A significant number of patients will still require postoperative radiotherapy following radical prostatectomy for certain pathologic high-risk features. Recently, three separate studies have demonstrated that adjuvant radiotherapy following radical prostatectomy improves disease control (biochemical progression-free survival), and Thompson et al. reported a marked overall survival benefit for high-risk patients following radical prostatectomy [53–56]. Furthermore, aforementioned studies by Spahn et al. and Walz et al. have documented that men with high-risk disease do not have uniformly poor outcomes after undergoing radical prostatectomy. Indeed, there is a subset of patients within this risk group that derive a comparative benefit from surgery, and thus RP should remain a genuine consideration for therapeutic intervention [11, 12, 57].

## 4. Chemotherapy and Prostate Cancer

### 4.1. The Use of Chemotherapy in Castration-Refractory Prostate Cancer

Although long-term ADT plus radiotherapy is currently the standard care for high-risk patients, many high-risk prostate malignancies still recur. Importantly, a proportion of these high-risk prostate tumors will become refractory to hormonal therapies which place the patient at risk of developing recurrent or metastatic disease [36, 58, 59]. Strategies to enhance the therapeutic benefits of treatment and improve survival outcomes have been studied, with a particular emphasis on systemic treatment such as chemotherapy. Castration-refractory metastatic prostate cancer patients were the first group of patients in which the efficacy of chemotherapy was assessed. The CALBG 9182 study examined a combination of mitoxantrone and hydrocortisone versus hydrocortisone alone. While there was no survival benefit reported among the 242 men with castration-refractory disease, a delay in disease progression and time to treatment failure was observed. Additionally, this study also confirmed the palliative benefits of this regimen previously reported in a small randomized trial in Canada [60, 61]. The CALBG 9182 study generated interest in exploring other chemotherapeutic agents in this population. 

Two prospective phase III trials for men with metastatic castration-refractory prostate cancer helped to establish docetaxel and prednisone as the preferred chemotherapy regimen. SWOG 9916, a prospective trial randomizing 674 men with castration-refractory disease, compared survival outcomes and toxicity profiles in a head-to-head comparison of a docetaxel plus estramustine versus mitoxantrone. The docetaxel-containing regimen demonstrated a significant increase in overall survival of nearly two months; however, there was also an increase in side effects, including neutropenic fever and cardiovascular events [62]. Subsequently, it has been reported that the addition of estramustine to docetaxel has been shown to increase side effects without enhancing efficiency [63]. The second pivotal study was the TAX 327 trial which compared docetaxel and mitoxantrone; prednisone was also administered in both regimens. Importantly, estramustine was not a component of the docetaxel-containing regimens and docetaxel was given in a weekly or in an every three-week schedule. Patients in the docetaxel every three-week arm demonstrated an improved median survival of 2.5 months with a 24% reduction in risk of death [64]. The 10-year update of this study reported continued survival benefit in the docetaxel every three-week arm [65]. 

### 4.2. The Role for Chemotherapy in the Management of High-Risk Localized Disease

These studies in metastatic castration-refractory patients helped lay the groundwork for early use of docetaxel and other agents as part of the primary treatment for high-risk and locally advanced prostate cancer patients (Table 4). The rationale of using chemotherapy and other systemic agents in the adjuvant setting is that micrometastatic disease as well as androgen-resistant clones will encounter cytotoxic treatment earlier [17]. As radiotherapy techniques permit increasing dose escalation, chemotherapy can play a more important synergistic role by radiosensitizing tumor cells at the primary site while also addressing micrometastatic disease. Specifically, docetaxel, a radiosensitizing cytotoxic antimicrotubule agent has been used extensively in the treatment of breast, ovarian, and nonsmall-cell lung cancer [66–68]. It exerts a direct cytotoxic effect by arresting cells in M phase, thereby preventing cell division. Furthermore, by stabilizing the cells in M phase, a radiosensitive phase of the cell cycle, docetaxel is able to synergize the effect of radiation [69–71]. 

A critical phase III multicenter study by Rosenthal et al. highlighted the severe toxicities that may occur with multichemotherapy multimodal regimens. A total of 397 high-risk non-metastatic patients (PSA 20–100 ng/mL and Gleason score ≥ 7 or stage > T2, Gleason score 8 and PSA < 100 ng/mL) enrolled in RTOG 99-02 and were randomized to an ADT plus radiotherapy with four cycles of adjuvant paclitaxel, estramustine and oral etoposide (TEE) group, or an ADT plus radiotherapy alone group. After opening in 2000, the trial was closed after 4 years due to excess thromboembolic events and severe toxicities, particularly in the adjuvant chemotherapy arm. With regards to short-term toxicities, 71% (136/192) of patients in the adjuvant TEE plus ADT and radiotherapy cohort reported grade 3 or greater toxicities compared with only 37% in the radiotherapy and ADT cohort. There was a significant increase in hematologic and gastrointestinal toxicity but not genitourinary toxicity. Furthermore, in terms of long-term complications, three cases of myelodysplasia/acute myelogenous leukemia were noted [14]. A follow-up prospective phase III trial, RTOG 05-21, was designed to assess the efficacy of a less toxic adjuvant chemotherapy regimen when combined with ADT and radiotherapy. This ongoing study compares high-risk patients receiving ADT (LHRH agonist and oral antiandrogen) and radiation (3D-CRT or IMRT) with or without adjuvant docetaxel chemotherapy. Enrollment criteria consists of: (1) Gleason score ≥ 9, PSA ≤ 150 ng/mL and any T stage disease (2) Gleason score 8, PSA < 20 ng/mL, stage T2 disease or higher (3) Gleason score 7-8, PSA 20–150 ng/mL, any T stage [15]. The results of this trial will certainly help to elucidate the role of chemotherapy in the adjuvant setting for high-risk patients.

Kumar et al. conducted a phase I trial of concurrent weekly docetaxel with 3D-CRT in order to discern to maximal tolerated dose (MTD) of weekly docetaxel for patients with unfavorable localized prostate cancer. The 22 patients who were enrolled in the concurrent docetaxel and 3D-CRT regimen met inclusion criteria of: (1) T3-T4 disease (2) T1b-T2 disease and Gleason score ≥ 8 or (3) T1c-T2 disease with Gleason score 5–7 and PSA ≥ 10. The MTD of weekly docetaxel was determined to be 20 mg/m^2^ in conjunction with 3D-CRT, in general, this regimen was considered to be well tolerated without any excessive or objectionable toxicity. Other relevant findings from the study include the side effect of grade 3 diarrhea which was the dose-limiting toxicity; however, no hematologic adverse side effects were noted [72]. Another small phase I study involving concurrent docetaxel and radiotherapy supported Kumar's findings of permissible toxicity with radiosensitizing regimens. The AGUSG 03-10 phase I/II prospective trial was a continuation upon Kumar et al.'s earlier work with docetaxel and 3D-CRT. In this study, concurrent IMRT was given with the previously determined MTD of weekly docetaxel at 20 mg/m^2^. Twenty high-risk patients with at least stage T3 disease, a Gleason score of 8–10 and PSA > 10 ng/mL were enrolled on this concurrent chemoradiation protocol. In general, the concurrent IMRT with weekly docetaxel regimen was well tolerated with acceptable toxicity. Furthermore, 85% of the patients were free of biochemical disease recurrence at a mean followup of 11.7 months. Of note, there were no grade 3 or 4 toxicities reported and the most common toxicities were grade 2 fatigue (40%), grade 2 diarrhea (40%), and grade 2 urinary frequency (35%) [73]. The two aforementioned studies highlighted the emerging role that concurrent taxane-based chemotherapy may play in the management of high-risk prostate cancer.

Sanfilippo et al. conducted a prospective phase I/II study of biweekly paclitaxel and concurrent radiotherapy in order to determine the maximum tolerated dose of paclitaxel in androgen-ablated locally advanced prostate cancer. Paclitaxel, a taxane molecule, has a similar mechanism of action to docetaxel which leads to the accumulation of cells in G2/M phase having both cytotoxic as well as radiosensitizing effects [80, 81]. This study involved 22 patients who had T2–T4 tumors with Gleason scores > 7 and/or PSA levels > 10 ng/mL and/or pathologic staging of TxN1. Patients underwent 3D-CRT with doses ranging from 63–73.8 Gy. It was concluded that concurrent biweekly paclitaxel with 3D-CRT is feasible with an MTD for combined paclitaxel and 3D-CRT of 73.8 Gy. Four patients developed grade 3 diarrhea, three at a dose of 66.6 Gy and one at the MTD of 73.8 Gy. With regards to patient outcomes, 21 of 22 patients (95%) were still alive and 6 of 22 (27%) patients experienced biochemically recurrent disease at a median followup of 28 months [74]. Importantly, in comparison to the aforementioned studies involving concurrent radiotherapy with paclitaxel, patients in this trial also received ADT in addition to concurrent chemoradiotherapy and the toxicities were not prohibitive.

Examining the efficacy of chemotherapy in the neoadjuvant setting in combination with ADT and radiotherapy is becoming an area of increasing interest. Hussain et al. evaluated the safety of neoadjuvant docetaxel and estramustine chemotherapy alone in 21 patients with high-risk cancer defined as clinical stage T2b or greater, PSA > 15 ng/mL and/or a Gleason score of 8–10. Induction chemotherapy with docetaxel and estramustine was reported to be a well-tolerated and a feasible regimen for the high-risk cohort. Patients did experience grade 3 and 4 toxicities in the form of neutropenia in nine patients and deep vein thrombosis in two patients. Furthermore, the efficacy of this regimen in comparison to ADT remains unclear and its use in conjunction with other modalities was not evaluated [75]. However, a prospective randomized study in Japan assessed the safety and efficacy of neoadjuvant ADT plus estramustine phosphate (EMP) combined with 3D-CRT for patients with both intermediate and high-risk prostate cancer. A total of 39 patients were randomized into a neoadjuvant ADT alone group or neoadjuvant ADT with EMP group, both groups received 3D-CRT to a total dose of 70 Gy. The 4-year biochemical relapse-free survival was 61% in the group receiving combined chemotherapy and androgen ablation compared to the 49% in the group receiving only neoadjuvant ADT. Additionally, there were no severe toxicities reported leading the authors to conclude that the combination of ADT with EMP in the neoadjuvant setting appears to be better than neoadjuvant ADT alone. However, both regimens were unable to prevent biochemical failure and thus suggested additional adjuvant therapy, particularly, in the high-risk cohort with pretreatment PSA > 20 ng/mL [76]. 

Recently, the role of chemotherapy in conjunction with radical prostatectomy has also been examined. Similarly to RTOG 99-02, which has halted due to prohibitive toxicities, SWOG 9921, a randomized phase III trial, prematurely closed the chemotherapy plus ADT arm due to the development of acute myelogenous leukemias. In this study, 983 patients with high-risk features were randomized to receive adjuvant ADT with or without mitoxantrone in the postradical prostatectomy setting. This study underlined the importance of prospective trials to assess potential safety issues for patients. In this particular case, the risk of secondary malignancies associated with a mitoxantrone-containing adjuvant chemotherapy regimen was an unexpected problem [77]. Importantly, in 2011 Dorff et al. reported preliminary data from the SWOG S9921 study because of the potential implications for future prospective trial design. Among the 481 high-risk patients (Gleason ≥ 8, preoperative PSA > 15 ng/mL or both) receiving ADT-alone after RP, the estimated 5-year biochemical failure-free survival is 92.5% and the 5-year overall survival is 95.9%. In light of the favorable outcomes achieved with adjuvant ADT post-RP, this study highlights the difficulty in demonstrating that the addition of chemotherapy can improve upon currently available therapies given the extremely low rate of disease recurrence and PCSM [78]. Of note, the cancer and leukemia group B has initiated an ongoing phase III trial (CALGB 90203) in high-risk patients randomizing them to be treated with neoadjuvant estramustine and docetaxel followed by surgery or surgery alone [79].

## 5. New Approaches on the Horizon

The need to improve high-risk disease management has prompted the development of novel agents, several of which may be genuine contenders to impact disease outcomes in the future. Initial studies in castration-refractory disease have been useful in order to characterize the efficacy and safety of these potential therapeutic agents.

Sipuleucel-T (APC8015), a cancer vaccine, is an active cellular immunotherapy that stimulates a prostate cancer-specific T-cell immune response against prostatic acid phosphatase (PAP), an antigen expressed by approximately 95% of prostate cancer cells [82–84]. Specifically, autologous antigen-presenting cells (APCs) collected via leukapheresis undergo ex vivo stimulation with PA2024 (recombinant fusion protein of PAP and GM-CSF) and are subsequently infused into the patient to exert an immunogenic effect. Presumably, the interaction of T cells with the PA2024-activated APCs primes the T cells for their highly specific tumoricidal activity [85, 86]. A 2010 multicenter double-blind, placebo-controlled study by Kantoff et al. evaluated a total of 512 patients with metastatic castration resistant disease in order to compare the efficacy of sipuleucel-T to placebo. Sipuleucel-T was shown to prolong overall survival with a 22% relative reduction in risk of death (hazard ratio, 0.78) which prolonged median survival by 4.1 months, however, no change in the time to disease progression was noted. There were no excessive toxicities observed, however, an increase in chills, fever, and headache was associated with infusion of sipuleucel-T [87]. Sipuleucel-T was approved by the FDA in April 2010 for the treatment of asymptomatic or minimally symptomatic metastatic castration-refractory prostate cancer. The early findings with sipuleucel-T appear to be promising but its efficacy in conjunction with chemotherapy and potential use in earlier settings such as high-risk prostate cancer is yet to be determined.

While prostate cancer patients often initially derive benefit from ADT, a proportion of these patients will develop castration-refractory prostate cancer characterized by progression of disease despite castrate levels of circulating testosterone. The persistence of ligand-mediated androgen receptor signaling implicates extragonadal (prostate, adrenal, intratumoral) androgen production as a potential mechanism of resistance to ADT rather than an androgen-independent mechanism. Abiraterone acetate suppresses extragonadal androgen biosynthesis via inhibition of CYP17 (cytochrome P-450c17), an enzyme that has been shown to be over expressed in castration-refractory disease. In a recent 2011 phase III multicenter trial, de Bono and colleagues evaluated the efficacy of abiraterone in castration-refractory patients with progression after docetaxel treatment. Patients were randomized to receive prednisone with either abiraterone or placebo and there was a median follow-up time of 12.8 months among the 1195 enrolled patients. A clear survival benefit was reported in the abiraterone-prednisone group, with a 35.4% reduction in the risk of death compared to the placebo cohort (hazard ratio, 0.65) which translated into an increase in overall survival of 3.9 months (14.8 versus 13.9 months). Secondary end points including PSA progression, progression-free survival and PSA response rate also favored the patients whom received abiraterone. Of note, while both treatment cohorts received prednisone as part of the treatment regimen, steroid-related toxicities and side effects were more frequent among patients receiving the androgen biosynthesis inhibitor. However, the general consensus regarding this study is that abiraterone acetate plus prednisone is effective in prolonging overall survival with minimal increase in additional toxicities for patients with metastatic castration-refractory prostate cancer with progression after chemotherapy [88, 89].

A new therapy, MDV3100, targets androgen receptor-mediated treatment resistance with a distinct mechanism of action to that of abiraterone. While the concept of androgen-receptor antagonism is not a novel concept, MDV3100 is notable for its extremely high receptor-binding affinity, ability to induce tumor cell apoptosis and pure androgen receptor antagonism [90]. These characteristics ensure more effective androgen signaling blockade in comparison to other agents such as bicalutamide that have a relatively lower receptor binding affinity and may exhibit partial agonism at the androgen receptor; a potential cause of refractory disease. Although, this agent is not as far along as sipuleucel-T and abiraterone in terms of clinical testing, a phase 1-2 study by Scher et al. has generated cautious optimism regarding MDV3100. This multicenter dose-escalation trial enrolled 140 patients with progression metastatic castration-refractory disease in order to assess the safety and tolerability of MDV3100 as well as to establish the maximum tolerated dose. The maximum tolerated dose was determined to be 240 mg/day and the most predominant grade 3-4 treatment-related toxicity was dose-dependent fatigue. Of note, antitumor effects were appreciated at all doses. This study has helped to corroborate that persistent androgen-receptor signaling is veritable target for castration-refractory disease and further preclinical and clinical studies are need to decided whether MDV3100 can truly impact outcomes among prostate cancer patients [91].

## 6. Conclusion

While considerable progress has been made in the treatment of high-risk prostate cancer, there is a clear need to continue prospective randomized clinical trials in order to optimize treatments. Combination therapies involving radiotherapy, androgen deprivation therapy, surgery and chemotherapy have yielded varied success. Importantly, the combination of long-term ADT and radiotherapy and has been particularly successful and chemotherapy may have the potential further improve outcomes. As we continue to appreciate the additive and synergistic effects of multimodality therapy, we must also acknowledge the potential for additive toxicities.

## Figures and Tables

**Table 1:** Anatomic stage/prognostic groups, high-risk localized prostate cancer.

Group	T	N	M	PSA	Gleason
I	T1a–c	N0	M0	PSA < 10	Gleason ≤ 6
	T2a	N0	M0	PSA < 10	Gleason ≤ 6
	T1-2a	N0	M0	PSA X	Gleason X
IIA	T1a–c	N0	M0	PSA < 20	Gleason 7
	T1a–c	N0	M0	10 ≤ PSA < 20	Gleason ≤ 6
	T2a	N0	M0	10 ≤ PSA < 20	Gleason ≤ 6
	T2a	N0	M0	PSA < 20	Gleason 7
	T2b	N0	M0	PSA < 20	Gleason ≤ 7
	T2b	N0	M0	PSA X	Gleason X
**IIB **	**T2c **	**N0 **	**M0 **	**Any PSA **	**Any Gleason **
	**T1-2 **	**N0 **	**M0 **	**PSA ** ≥ ** 20 **	**Any Gleason **
	**T1-2 **	**N0 **	**M0 **	**Any PSA **	**Gleason > 8 **
**III **	**T3a-b **	**N0 **	**M0 **	**Any PSA **	**Any Gleason **
**IV **	**T4 **	**N0 **	**M0 **	**Any PSA **	**Any Gleason **
	Any T	N1	M0	Any PSA	Any Gleason
	Any T	Any N	M1	Any PSA	Any Gleason

Adapted from American Joint Committee on Cancer (AJCC Cancer Staging Manual, Seventh Edition, 2010).

**Table 2:** Prognostic factors for recurrence risk in localized prostate cancer.

Very low	Low	Intermediate	High	Very high (locally advanced)
T1c	T1-T2a	T2b-T2c	T3a	T3b-T4
Gleason < 6	Gleason 2–6	Gleason = 7	Gleason 8–10	
PSA < 10	PSA < 10	PSA 10–20	PSA > 20	
<3 (+) biopsy cores w/ ≤ 50% cancer per core				
PSA density < 0.15 ng/mL/g				

Adapted from NCCN Clincal Practice Guidelines in Oncology Prostate Cancer V.1.2011© 2011 National Comprehensive Cancer Network, Inc.

**Table 3:** Risk stratification for high-risk prostate cancer.

Source	High-risk definition
D'Amico et al. [13]	Stage T2c or PSA > 20 ng/mL or Gleason > 8
RTOG 9902, 0521 [14, 15]	Any T stage, PSA 20–100 ng/mL, Gleason > 7 or stage ≥ T2, PSA < 100 ng/mL, Gleason 8–10
NCCN (v1.2011) [16]	Stage ≥ T3 and/or PSA > 20 ng/mL and/or Gleason 8–10*

Adapted from Nat Rev Urol 2010 Nature Publishing Group [17]. *Combines high-risk and very high risk (locally advanced) groups.

**Table 4:** Summary of randomized control trials involving chemotherapy for high-risk localized prostate cancer.

Study (reference)	Chemo sequencing	Chemo regimen	Study arms	Number of patients	High-risk criteria		
					Stage	Gleason	PSA
RTOG 9902 [14]	Adjuvant	paclitaxel estramustinee toposide (TEE)	ADT + RT versus ADT + RT + TEE	397	any T	≥7	20–100
					≥ T2	8–10	<100

RTOG 0521 [15]	Adjuvant	Docetaxel	ADT + RT versus ADT + RT + docetaxel	612	any T	≥9	≤150
					≥ T2	8	<20
					any T	7-8	≥20–150

Kumar et al. [72]	Concurrent	Docetaxel	RT + docetaxel	22	T3-T4		
					T1b-T2	≥8	
					T1c-T2	5–7	≥10

AGUSG 03-10 [73]	Concurrent	Docetaxel	RT + docetaxel +/− ADT	20	≥T3	8–10	
						7	>10

Sanfilippo et al. [74]	Concurrent	Paclitaxel	ADT + RT versus ADT + RT + paclitaxel	22	TxN1	>7	>10

Hussain et al. [75]	Neoadjuvant	Docetaxel estramustine	docetaxel, EMP +/− RP, RT	21	≥T2b	8–10	≥15

Hirano et al. [76]	Neoadjuvant/ concurrent	Estramustine	ADT + RT versus ADT + RT + EMP	39	≥T3	8–10	>20

SWOG S9921 [77, 78]	Neoadjuvant	Mitoxantrone	RP + ADT versus RP + ADT + MTX	983	pT3b-T4	≥8	
						7	>15

CALGB 90203 [79]	Neoadjuvant	Estramustine docetaxel	RP versus EMP and docetaxel + RP	recruiting	T1-T3a, NX, M0		

ADT: androgen deprivation therapy; RT: radiotherapy; TEE: paclitaxel, estramustine, etoposide; EMP: estramustine phosphate; RP: radical prostatectomy; MTX: mitoxantrone.
